# Chronic-binge ethanol feeding aggravates systemic dyslipidemia in *Ldlr* ^-/-^ mice, thereby accelerating hepatic fibrosis

**DOI:** 10.3389/fendo.2023.1148827

**Published:** 2023-07-25

**Authors:** Constanze Hoebinger, Dragana Rajcic, Beatriz Silva, Tim Hendrikx

**Affiliations:** ^1^ Department of Laboratory Medicine, Medical University Vienna, Vienna, Austria; ^2^ Department of Biochemistry, Chemistry Institute, University of Sao Paulo, Sao Paulo, Brazil

**Keywords:** alcohol-associated liver disease (ALD), dyslipidemia, hypercholesterolemia, low-density lipoprotein receptor (LDLR), Ldlr-/- mice, alcoholic fibrosis, inflammation

## Abstract

**Objective:**

Chronic ethanol consumption is known to cause alcohol-associated liver disease, which poses a global health concern as almost a quarter of heavy drinkers develop severe liver damage. Alcohol-induced liver disease ranges from a mild, reversible steatotic liver to alcoholic steatohepatitis and irreversible liver fibrosis and cirrhosis, ultimately requiring liver transplantation. While ethanol consumption is associated with dysregulated lipid metabolism and altered cholesterol homeostasis, the impact of dyslipidemia and pre-existing hypercholesterolemia on the development of alcohol-associated liver disease remains to be elucidated.

**Design:**

To address the influence of systemic dyslipidemia on ethanol-induced liver disease, chronic-binge ethanol feeding was applied to female C57BL/6J (wild type) mice and mice deficient for the low-density lipoprotein receptor (*Ldlr^-/-^*), which display a human-like lipoprotein profile with elevated cholesterol and triglyceride levels in circulation. Respective control groups were pair-fed an isocaloric diet.

**Results:**

Chronic-binge ethanol feeding did not alter systemic lipid levels in wild type mice. While increased systemic cholesterol levels in *Ldlr^-/-^* mice were not affected by ethanol feeding, chronic-binge ethanol diet aggravated elevated plasma triglyceride levels in *Ldlr^-/-^* mice. Despite higher circulatory triglyceride levels in *Ldlr^-/-^* mice, hepatic lipid levels and the development of hepatic steatosis were not different from wild type mice after ethanol diet, while hepatic expression of genes related to lipid metabolism (*Lpl*) and transport (*Cd36*) showed minor changes. Immunohistochemical assessment indicated a lower induction of infiltrating neutrophils in the livers of ethanol-fed *Ldlr^-/-^* mice compared to wild type mice. In line, hepatic mRNA levels of the pro-inflammatory genes *Ly6g*, *Cd11b*, *Ccr2*, *Cxcl1* and *F4/80* were reduced, indicating less inflammation in the livers of *Ldlr^-/-^* mice which was associated with reduced *Tlr9* induction. While systemic ALT and hepatic MDA levels were not different, *Ldlr*-deficient mice showed accelerated liver fibrosis development after chronic-binge ethanol diet than wild type mice, as indicated by increased levels of Sirius Red staining and higher expression of pro-fibrotic genes *Tgfb*, *Col1a1* and *Col3a1*. *Ldlr^-/-^* and wild type mice had similar plasma ethanol levels and did not show differences in the hepatic mRNA levels of *Adh1* and *Cyp2e1*, important for ethanol metabolism.

**Conclusion:**

Our results highlight that chronic-binge ethanol feeding enhances systemic dyslipidemia in *Ldlr^-/-^* mice which might accelerate the development of hepatic fibrosis, independent of hepatic lipid levels.

## Introduction

Chronic consumption of ethanol-containing beverages has detrimental effects on tissue homeostasis and can enhance the development of various diseases in different organs, such as the liver. In fact, alcohol-associated liver disease is the most prevalent chronic liver disease worldwide and a leading cause of liver-related morbidity and mortality, and thus governs a dramatic socio-economic burden (1, 2). It encompasses a spectrum of pathologies ranging from simple steatosis (accumulation of triglycerides in hepatocytes) to alcohol-associated steatohepatitis (ASH), which is characterized by hepatic inflammation, hepatocyte injury, and ballooning (3). A persistent increase in triglyceride accumulation, inflammation, and liver injury stimulates fibrotic scarring in the liver. Subsequently, fibrosis may culminate in the development of end-stage liver diseases such as cirrhosis or hepatocellular carcinoma, ultimately requiring liver transplantation (4). Despite considerable progress in recent decades, the mechanisms involved in the pathogenesis and progression of alcohol-induced liver disease are not fully understood. The fact that up to 20% of heavy drinkers develop advanced fibrosis and cirrhosis, while for the remaining 80%, the progression of the disease is much slower, poses the need to identify the underlying factors that contribute to these variations (5).

Altered lipid metabolism and resulting dyslipidemia are key processes by which ethanol consumption contributes to the development of alcohol-related fatty liver disease (6). In the liver, it has been described that ethanol alters lipid metabolism by increasing hepatic fatty acid uptake and triglyceride synthesis through *de novo* lipogenesis (7). Furthermore, studies have indicated that ethanol impairs cholesterol synthesis by reducing the formation and secretion of very low density lipoprotein (VLDL). Since VLDL gets assembled in the liver from triglycerides, cholesterol, and apolipoproteins and enables lipids to enter the circulation, a decreased cholesterol synthesis promotes the storage of triglycerides as lipid droplets in the liver (8–11). Yet, some studies have reported opposite effects, namely increased hepatic cholesterol synthesis (12, 13). In parallel with altered hepatic lipid metabolism, heavy drinking is associated with systemic dyslipidemia, characterized by elevated blood low-density lipoprotein cholesterol (LDL-C), high triglyceride levels, and low levels of high-density lipoprotein cholesterol (HDL-C) (14, 15). As such, it was reported that daily moderate alcohol intake poses a risk factor for hypertriglyceridemia, whereas it was shown to be protective in terms of developing hypercholesterolemia (15). However, after binge drinking, elevated cholesterol levels were measured in humans and rats (13, 16). Despite these divergent observations, these data indicate that ethanol abuse might have important implications for diseases such as atherosclerosis, for which alterations in systemic lipid levels are established risk factors (17). While it is well-documented that alcoholic hepatitis patients have an altered lipid metabolism, the contribution of pre-existing systemic dyslipidemia to the development and progression of alcohol-associated liver disease is currently unclear. Therefore, we assessed ethanol-induced steatohepatitis in mice lacking the low-density lipoprotein receptor (*Ldlr^-/-^*), which resemble the human lipoprotein profile and are characterized by elevated cholesterol and triglyceride levels in circulation due to defective LDL clearance (18).

## Materials and methods

### Animal experiments

C57BL/6J mice were purchased from Charles River. *Ldlr^-/-^* mice (stock no. 002207) were originally purchased from The Jackson Laboratory and were acquired for this study from our in-house breeding facility at the Medical University of Vienna, Austria. All mice were on a C57BL/6J background and were maintained in the specific pathogen-free (SPF) facility. Mice were bred under barrier-specific pathogen-free conditions at the Department of Biomedical Research or the Department of Laboratory Animal Science and Genetics of the Medical University of Vienna, Austria, and housed in individually-ventilated cages with a 12-hour dark-/light-cycle with *ad libitum* access to liquid diet. All female mice used in the experiments were aged 9 to 10 weeks. All experimental studies, interventions, and sample sizes were approved by the Animal Ethics Committee of the Medical University of Vienna and the Austrian Federal Ministry of Education, Science and Research and were performed according to Good Scientific Practice guidelines (License number: 2022-0.574.136).

### Dietary interventions

For chronic-binge ethanol feeding, an adapted protocol (19) of the NIAAA model was applied (20). Mice were fed Lieber–DeCarli diet for 15 days starting at day 6 with ethanol feeding. The caloric intake from EtOH was 0 on days 1–5 and 36% (6.4% (v/v)) from day 6 until the end. At day 16, mice were gavaged with one dose of ethanol (5 g/kg BW) and sacrificed 8 hours later. Pair-fed control mice received a diet with an isocaloric substitution of dextrose. The liquid diet was freshly prepared 3 times/week with an irradiated diet and was administered *ad libitum* in the ethanol-fed groups. The sacrifice of experimental mice occurred randomly with alternating order of genotype to prevent confounding effects of time of harvest. For all further analysis, measurements were done randomly and in a blinded fashion.

### Biochemical analyses

Blood was collected in EDTA collection tubes (Greiner Bio-One, Germany), and plasma was obtained by centrifugation at 2000xg for 10 minutes. ALT plasma levels were determined using Reflotron ALT strips on a Reflotron Plus (Roche). Plasma and hepatic triglyceride and cholesterol levels were measured according to the manufacturer’s instructions using Liquid Reagents kit (GPO-PAP Triglyceride Liquicolor kit, CHOD-PAP Cholesterol Liquicolor kit, HUMAN Biochemica, and Diagnostica mbH, Wiesbaden, Germany). Protein content was measured using the Pierce BCA Protein Assay Kit (Thermo Fisher Scientific, Waltham, MA, USA). TBARS were assessed following the manufacturer’s instructions using the Quantichrome DTBA-100 kit (BioAssay Systems, USA). Plasma ethanol levels were determined using the Ethanol assay kit (MAK076-1KT, Sigma-Aldrich, Vienna, Austria) according to the manufacturer’s protocol.

### Immunohistochemistry

Mouse liver sections were embedded in OCT compound, and 7 μm frozen sections were stained and quantified for CD11b (Mac-1; Clone: M1/70; 550282, Becton Dickinson, Austria; 1:1000) for infiltrating macrophages and neutrophils, and with Oil Red O (Sigma-Aldrich, Vienna, Austria) to determine lipid content as reported previously (21). Formalin-fixed liver samples were embedded in paraffin, and 4 μm sections were stained for hematoxylin and eosin for liver morphology and steatosis and with Sirius Red for liver fibrosis detection as described previously (22). Recorded images were analyzed for the hepatic collagen content using ImageJ 1.53 software and quantified for the percentage of positive staining per liver section. At least 3 randomly selected images of the liver per mouse were analyzed. In addition, an arbitrary scoring of Sirius red staining of whole liver sections was done by an experienced experimental hepatologist. Furthermore, formalin-fixed liver samples were stained and quantified for Ly6G+ infiltrating neutrophils (Rat anti-mouse Ly6G IgG2b; NIMP-R14; MA1-40038; 1:700; ThermoFisher). In short, 4 μm thickness sections were deparaffinized and rehydrated with xylene, decreasing ethanol concentrations, and distilled water. Antigen retrieval was performed by incubating the sections in Citrate Buffer pH 6.0 at 95°C. Sections were incubated with the primary antibody overnight at 4°C. Biotinylated goat anti-rat IgG (BA-9401; VectorLABS) was used as secondary antibody. The colour was developed with diaminobenzidine, and the nuclei were counterstained with hematoxylin, followed by dehydration with increasing ethanol concentrations. The quantification of positive cells was carried out by averaging the number of positively-stained cells from 5 randomly selected high-power fields of the liver per mouse.

### RNA isolation

For whole liver RNA isolation, 50mg tissue pieces of left lateral liver lobes were snap-frozen in liquid nitrogen. Frozen tissue in QIAzol lysis reagent was homogenized mechanically using 1.0 mm TriplePure M-Bio Grade High Impact Zirconium beads (Lot:44544432, Benchmark) in a Tissue Lyser II (Qiagen, Hilden, Germany). RNA was extracted by QIAzol (QIAzol lysis reagent; Cat. No./ID: 79306; Qiagen, Hilden, Germany) according to the manufacturer’s instructions. RNA content and quality were assessed using Nanodrop (Peqlab).

### cDNA generation and qPCR

For quantitative real-time PCR, up to 1μg of RNA was reverse transcribed using the High Capacity cDNA Reverse Transcription kit (Applied Biosystems, Thermo Fisher, Waltham, MA, USA) to generate cDNA. Real-time PCR was performed on a CFX96 Real-Time PCR System (Bio-Rad Laboratories, Hercules, CA, USA) using the KAPA SYBR FAST kit (Thermo Fisher, Waltham, MA, USA) and the primers indicated below. Gene expression was calculated using the 2^–ΔΔCt^ method and normalized to the expression of housekeeping gene *18S*.

**Table:** 

Gene	Forward sequence 5’-3’	Reverse sequence 5’-3’
*18S*	AGTCCCTGCC CTTTGTACACA	CGATCCCAGG GCCTCACTA
*Cxcl1*	GCTGGGATTC ACCTCAAGAA	TCTCCGTTA CTTGGGGACAC
*Cxcl2*	AGTGAACTG CGCTGTCAATG	TTCAGGGTC AAGGCAAACTT
*Ccr2*	CAGGTGACAGAG ACTCTTGGAATG	GAACTTCTCTCCAACA AAGGCATAA
*Cd11b*	ATGGACGCTG ATGGCAATACC	TCCCCATTCA CGTCTCCCA
*Adh1*	GGGTTCTCA ACTGGCTATGG	ACAGACAGAC CGACACCTCC
*Cyp2e1*	CTTAGGGA AAACCTCCGCAC	GGGACATTCC TGTGTTCCAG
*Col1a1*	AACCCTGC CCGCACATG	CAGACGGCTG AGTAGGGAACA
*Tgfb*	AGCGCTCAC TGCTCTTGTGA	GTCGCCCCG ACGTTTG
*Ly6G*	GGCTCAGAA AAGTGCACCA	CGTACGTGG AAGCGAACAG
*Srebp1c*	GGAGCCATG GATTGCACATT	GCTTCCAGAG AGGAGGCCAG
*Srebpf2*	GCGTTCTGG AGACCATGGA	ACAAAGTTG CTCTGAAAACAAATCA
*Lpl*	GGGAGTTTG GCTCCAGAGTTT	TGTGTCTTCAG GGGTCCTTAG
*Fas*	AAGTTGCCC GAGTCAGAGAACC	ATCCATAGAGC CCAGCCTTCCATC
*ApoE*	CAGAGCTCC CAAGTCACACA	TGTGTGACTT GGGAGCTCTG
*Abca1*	GGTTTGGAGA TGGTTATACAATAGTTGT	CCCGGAAACG CAAGTCC
*Abcg1*	TCACCCAGTT CTGCATCCTCTT	GCAGATGTGT CAGGACCGAG
*Cd36*	GCCAAGCTAT TGCGACATGA	AAAAGAATCT AATGTCCGAGACTTT
*Sra1*	CATACAGAAA CACTGCATGTCAGAGT	TTCTGCTGATA CTTTGTACACACGTT
*Tlr2*	AAGAGGAAGC CCAAGAAAGC	CGATGGAATC GATGATGTTG
*Tlr4*	TATCCAGGTGTGAA ATTGAAACAATT	GGGTTTCCTGTC AGTATCAAGTTTG
*Tlr9*	ACTGAGCACC CCTGCTTCTA	AGATTAGTCA GCGGCAGGAA
*F4/80*	CTTTGGCTATG GGCTTCCAGTC	GCAAGGAGGAC AGAGTTTATCGTG
*Col3a1*	GACCAAAAGGT GATGCTGGACAG	CAAGACCTC GTGCTCCAGTTAG

### Statistical analysis

Statistical analyses were performed using GraphPad Prism v9.1.2. Our data comprised four groups: C57BL/6J mice (ethanol feeding/isocaloric controls) and *Ldlr^-/-^* mice (ethanol feeding/isocaloric controls). Comparison of the four groups was conducted using one-way analysis of variance (ANOVAs), and significant main effects were followed up by Fisher’s least significant difference (LSD) tests to compare individual groups and conditions. In outcomes with additional pre-post comparisons, we analyzed the data using factorial ANOVAs (with the factors group and time). Significant main effects or interactions were followed up again by LSD tests. Results are expressed as mean ± standard error (SEM) unless stated otherwise. A *p ≤* 0.05 was considered statistically significant.

## Results

### Ethanol feeding leads to increased plasma triglyceride levels in *Ldlr^-/-^* mice compared to wild type mice

**Figure 1:**
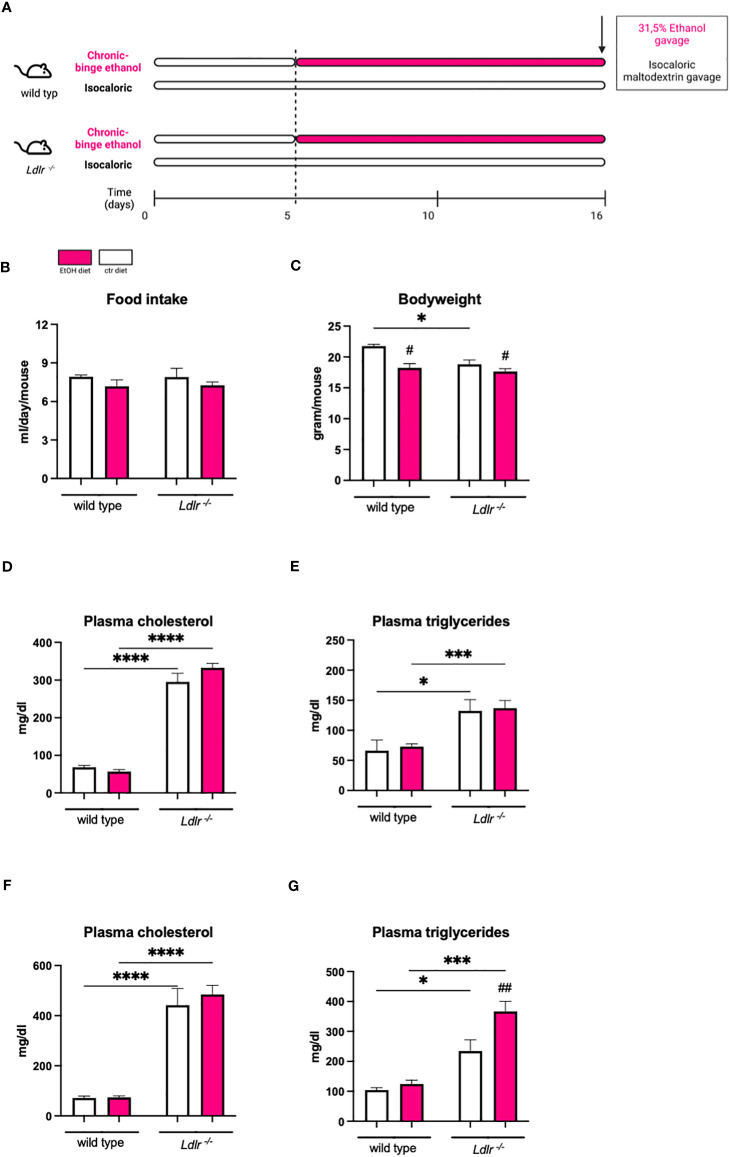
Chronic-binge ethanol feeding results in elevated plasma triglyceride levels in *Ldlr^-/-^* compared to wild type mice. **(A)** Schematic depiction of 15 days Lieber-DeCarli ethanol diet or isocaloric control diet feeding study with a single gavage of ethanol or isocaloric maltodextrin on day 16, respectively, in female C57BL/6J and female *Ldlr^-/-^* mice. Organs were harvested 8 hours after binge. **(B)** Amount of food intake in ml/day/mouse. **(C)** Body weight/mouse in grams at the end of the study. **(D)** Plasma cholesterol levels in mg/dl at the start of the diet intervention. **(E)** Plasma triglyceride levels in mg/dl at the start of the diet intervention. **(F)** Plasma cholesterol levels in mg/dl at the study endpoint. **(G)** Plasma triglyceride levels in mg/dl at the study endpoint. Data shown as mean ± SEM of n=4-17 mice/group. * indicates significant differences between wild type C57BL/6J mice and *Ldlr^-/-^* mice. # indicates significant differences between isocaloric control-fed and ethanol–fed mice. * indicates p ≤0.05, *** p≤0.001, **** p≤0.0001. # indicates p ≤0.05, ## indicates p≤0.01.

To investigate the contribution of pre-existing dyslipidemia to ethanol-induced liver disease, age-matched female *Ldlr^-/-^* mice and wild type (C57Bl6J) mice were subjected to a chronic-binge ethanol feeding model (19). Respective control mice were pair-fed an isocaloric control diet (**Figure 1A**). While the amount of food intake was similar between all experimental groups, ethanol-fed mice had a slightly lower body weight than control-fed mice, irrespective of the genotype. No difference in body weight between wild type and *Ldlr^-/-^* mice on ethanol diet was observed (**Figures 1B, C**). To assess whether ethanol consumption affects systemic lipid levels during dyslipidemia, plasma cholesterol and triglyceride levels were measured at the beginning and the end of the study. As expected, mice lacking the LDLR had significantly higher plasma cholesterol and triglyceride levels compared to wild type mice at baseline (**Figures 1D, E**). While *Ldlr^-/-^* mice had higher plasma cholesterol levels than wild type mice, no additional effect of ethanol feeding was observed in either genotype (**Figure 1F**). In contrast, *Ldlr^-/-^* mice showed increased plasma triglyceride levels after ethanol feeding compared to their respective pair-fed controls, while the amount of systemic triglycerides of wild type mice was not affected after chronic-binge ethanol feeding (**Figure 1G**). These data indicate that ethanol consumption exaggerates elevated plasma triglyceride levels during dyslipidemia in *Ldlr*-deficient mice.

### Hepatic lipid levels are similar between wild type and *Ldlr^-/-^* mice after ethanol feeding

**Figure 2:**
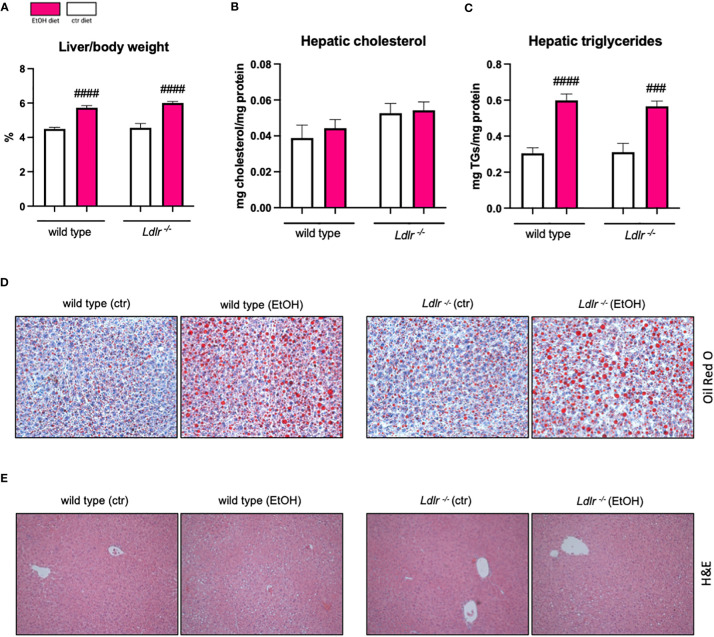
Wild type and *Ldlr^-/-^* mice develop hepatic steatosis to the same extent following chronic-binge ethanol feeding. **(A)** Liver to body weight ratio in percentage.**(B)** Hepatic cholesterol levels normalized to liver protein content. **(C)** Hepatic triglyceride levels normalized to liver protein content. **(D)** Representative images showing Oil Red O staining of liver sections. (magnification 20x) **(E)** Representative images showing H&E staining of liver sections. (magnification 10x) Data shown as mean ± SEM of n=4-17/group. # indicates significant differences between isocaloric control-fed and ethanol–fed mice. # indicates p ≤0.05, ### indicates p≤0.001, #### indicates p≤0.0001.

To determine to which extent ethanol intake during *Ldlr*-deficiency affects liver steatosis, hepatic cholesterol and triglyceride levels were measured. While ethanol diet did not alter cholesterol levels in the liver, chronic-binge ethanol feeding resulted in increased liver weight and the accumulation of hepatic triglycerides in *Ldlr^-/-^* and wild type mice compared to their respective pair-fed controls (**Figures 2A–D**). Although *Ldlr^-/-^* mice display altered systemic lipid levels, no differences in the amount of hepatic cholesterol and triglyceride content were observed compared to wild types, which was confirmed by Oil Red O staining (**Figures 2B–D**). In addition, no major morphological differences were observed in livers of *Ldlr^-/-^* and wild type mice after ethanol feeding (**Figure 2E**). These data indicate that systemic dyslipidemia due to the absence of the LDLR does not alter the degree of hepatic steatosis development in mice fed the chronic-binge ethanol diet.

**Figure 3:**
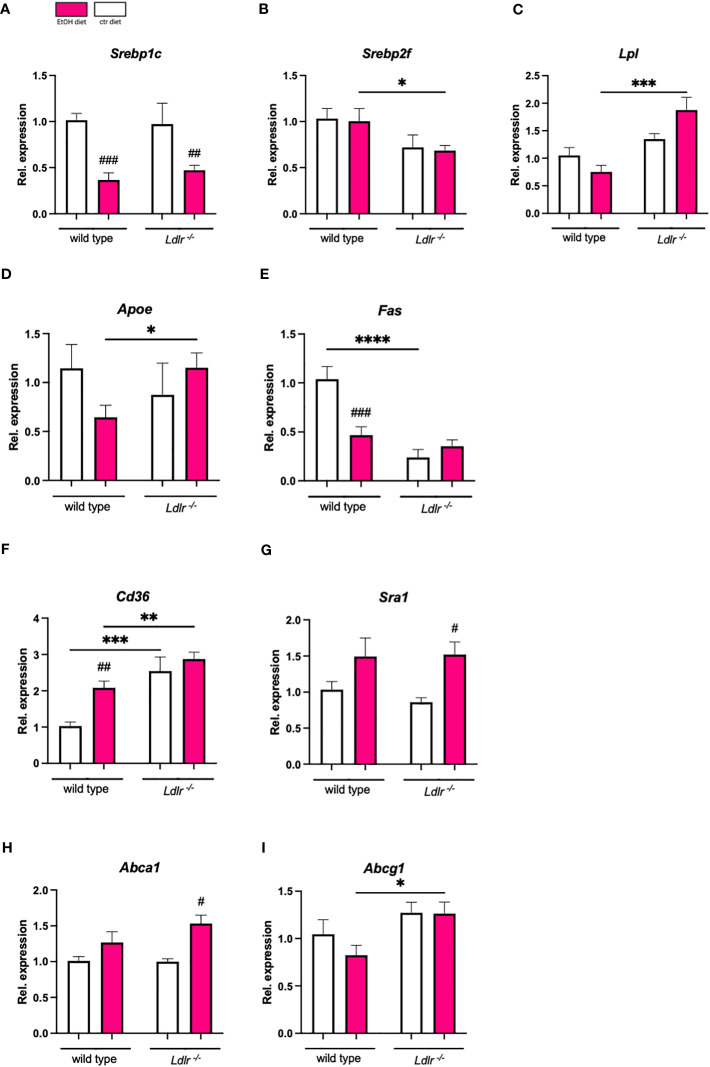
Hepatic expression of genes related to lipid metabolism and transport show minor changes between wild type and *Ldlr^-/-^* mice. **(A-I)** mRNA levels of indicated genes in livers of ethanol- and control-fed female C57BL/6J and *Ldlr^-/-^* mice, assessed by qPCR. Data are shown relative to the wild type isocaloric control-fed mice and normalized to *18S*. Data shown as mean ± SEM of n=4-17/group. # indicates significant differences between isocaloric control-fed and ethanol–fed mice. * indicates p ≤0.05, ** p≤0.01, *** p≤0.001, **** indicates p≤0.0001. # indicates p ≤0.05, ## p≤0.01, ### p≤0.001.

Next, we assessed hepatic mRNA levels of genes related to lipid metabolism and uptake in the liver, which might explain the observed accelerated hypertriglyceridemia in *Ldlr^-/-^* mice. While expression of *Srebp1c*, a gene involved in *de novo* lipogenesis, was downregulated after chronic-binge ethanol feeding in both genotypes, *Srebpf2*, which is crucial for cholesterol synthesis, remained unaffected by ethanol intake (**Figures 3A, B**). Notably, hepatic levels of *Lpl* mRNA were significantly higher in *Ldlr^-/-^* mice following ethanol administration than in wild type mice, while no genotype-related differences in *Fas* and *ApoE* gene expression following ethanol exposure were detectable. However, *Fas* and *ApoE* were downregulated in response to ethanol wild type but not in *Ldlr^-/-^* mice (**Figures 3C–E**), indicating altered lipid metabolism during *Ldlr*-deficiency. Interestingly, while expression of *Sra1* did not differ after chronic-binge ethanol feeding, *Ldlr^-/-^* mice displayed increased hepatic expression of *Cd36*, important for uptake of oxidized LDL (**Figures 3F, G**). Further, while ethanol diet only minor affected cholesterol efflux *via* the expression of *Abca1* and *Abcg1*, *Ldlr^-/-^* mice had higher *Abcg1* mRNA levels in the liver after chronic-binge ethanol consumption compared to wild type mice (**Figures 3H, I**). Taken together, while expression levels of genes related to *de novo* lipogenesis seem not affected by the lack of the LDLR during ethanol consumption, increased *Cd36* expression levels in mice deficient for *Ldlr* might enhance uptake of oxidized lipids, thereby triggering an inflammatory response in the liver.

### *Ldlr^-/-^* mice have less hepatic neutrophil recruitment compared to wild type mice after ethanol diet

**Figure 4:**
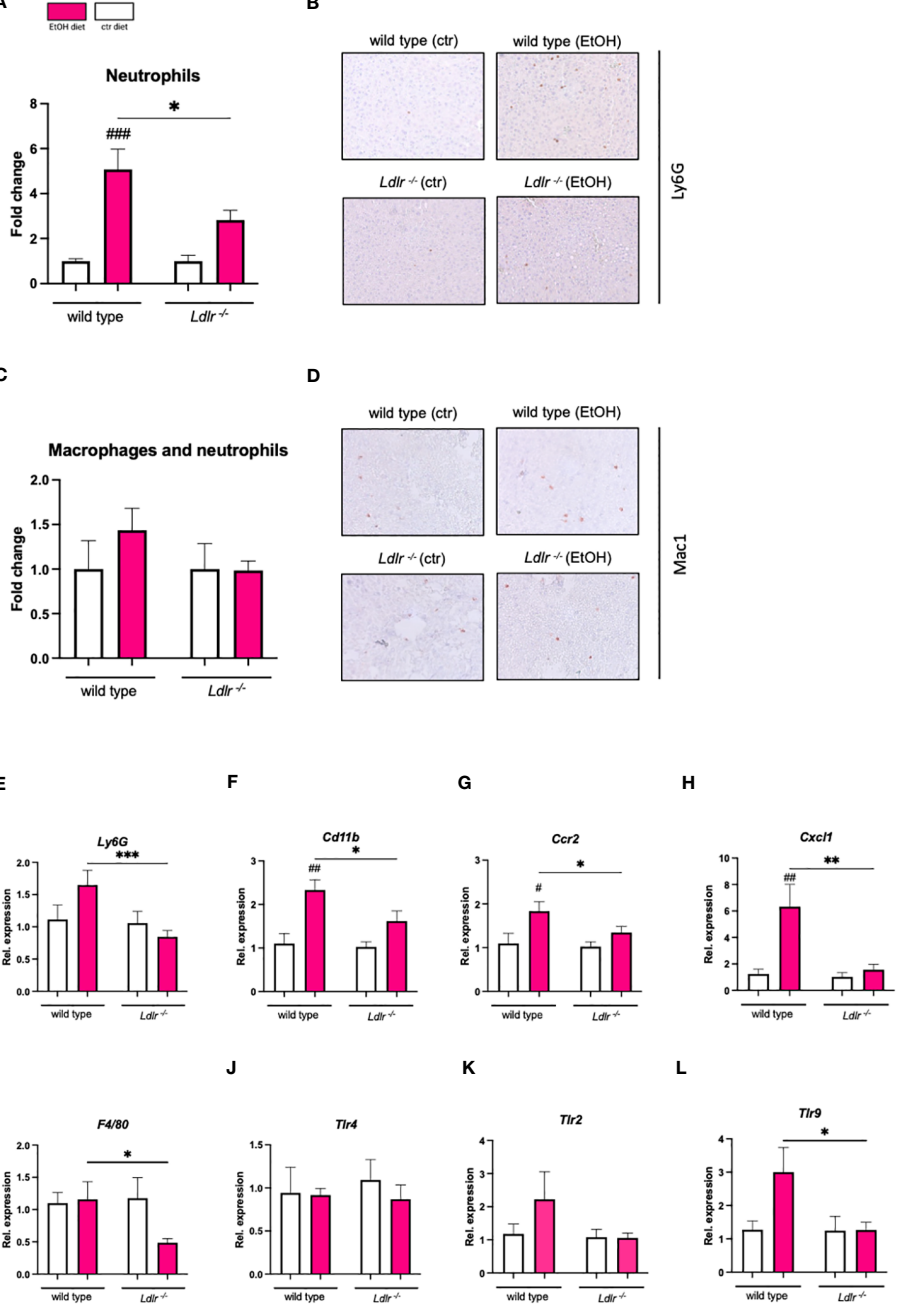
*Ldlr^-/-^* mice exhibit reduced hepatic inflammation compared to wild type mice following chronic-binge ethanol feeding. **(A)** Fold change of infiltrating neutrophils in the liver, assessed and quantified by immunohistochemical staining for NIMP. **(B)** Representative pictures of NIMP staining. (magnification 20x) **(C)** Fold change of infiltrating macrophages and neutrophils in the liver, assessed and quantified by immunohistochemical staining for Mac-1. **(D)** Representative pictures of Mac-1 staining. (magnification 20x) **(E-L)** mRNA levels of indicated genes (*Ly6g, Cd11b, Ccr2, Cxcl1, F4/80, Tlr2, Tlr4, Tlr9*) in livers of ethanol- and control-fed female C57BL/6J and *Ldlr^-/-^* mice, assessed by qPCR. Data are shown relative to the respective isocaloric control-fed mice and normalized to *18S*. Data shown relative to the respective isocaloric control-fed mice as mean ± SEM of n=3-16/group. * indicates significant differences between wild type C57BL/6J mice and *Ldlr^-/-^* mice. # indicates significant differences between isocaloric control-fed and ethanol–fed mice.* indicates p ≤0.05, ** p≤0.01, *** p≤0.001. # indicates p ≤0.05, ## p≤0.01, ### p≤0.001.

Next, we determined whether elevated plasma lipid levels in mice lacking the LDLR affect ethanol-induced hepatic inflammation. Since alcohol-associated liver disease is characterized by the influx of inflammatory cells, particularly neutrophils (23), liver sections of *Ldlr^-/-^* and wild type mice were stained for infiltrating neutrophils and macrophages. In line with our expectations, chronic-binge ethanol feeding resulted in a significant induction in the recruitment of Ly6G^+^ neutrophils (NIMP^+^) to the livers of wild type mice (**Figures 4A, B**). A similar trend was observed upon immunohistochemical assessment of CD11b^+^ macrophages and neutrophils (Mac-1) in the livers of wild type mice (**Figures 4C, D**). Surprisingly, chronic-binge ethanol feeding resulted in less recruitment of neutrophils and macrophages to the livers in *Ldlr^-/-^* mice compared to pair-fed control mice (**Figures 4A–D**). In agreement with our histological observations, we found that the hepatic induction of pro-inflammatory genes *Ly6g*, *Cd11b*, *Ccr2, Cxcl1*, and *F4/80*, are significantly lower in *Ldlr^-/-^* mice than in wild type mice after ethanol diet (**Figures 4E–H**). Furthermore, we measured the gene expression levels of Toll-like receptors that are involved in regulating inflammatory responses. While *Tlr4* was unchanged, mRNA levels of *Tlr2* and *Tlr9* were upregulated by ethanol intake in wild type controls (**Figures 4J–L**). Yet, *Ldlr^-/-^* mice failed to induce the expression of *Tlr2* and *Tlr9* after chronic-binge ethanol feeding. These data indicate that chronic-binge ethanol feeding in *Ldlr^-/-^* mice induces less neutrophil recruitment and inflammation in the liver compared to wild type mice, potentially as a result of diminished TLR signaling pathways.

### Despite similar degrees of liver injury, *Ldlr^-/-^* mice seem to be more prone to develop hepatic fibrosis than wild type mice after chronic-binge ethanol feeding

**Figure 5:**
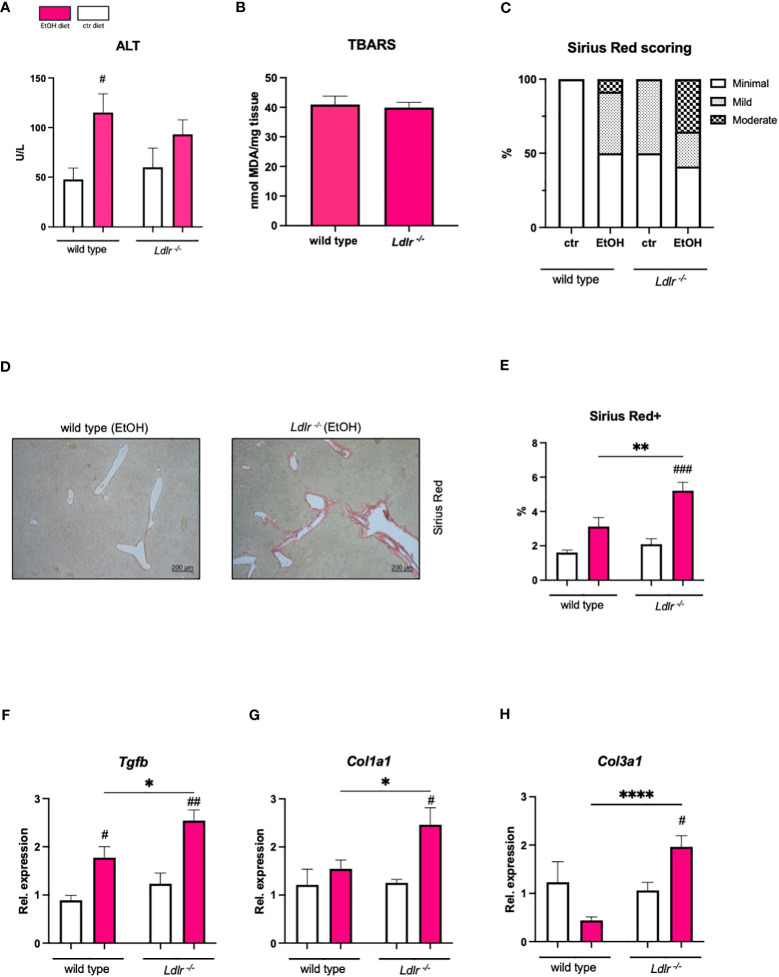
Despite comparable liver damage, *Ldlr^-/-^* mice exhibit increased susceptibility to ethanol-induced liver fibrosis compared with wild type mice. **(A)** Plasma ALT levels. **(B)** TBARS assay for MDA in liver tissue. **(C)** Percentage of mice/group with indicated arbitrary scoring for liver fibrosis based on Sirius red staining. **(D)** Representative pictures of Sirius Red staining of liver sections of ethanol-fed mice. (magnification 5x) **(E)** Quantification of immunohistochemical Sirius Red staining for liver fibrosis. **(F–H)** mRNA levels of indicated genes (*Tgfb, Col1a1, Col3a1*) in livers of ethanol- and control-fed female C57BL/6J and female *Ldlr^-/-^* mice, assessed by qPCR. Data are shown relative to the isocaloric control-fed wild type mice and normalized to *18S*. Data shown as mean ± SEM of n=5-17/group. * indicates significant differences between wild type C57BL/6J mice and *Ldlr^-/-^* mice. # indicates significant differences between isocaloric control-fed and ethanol–fed mice. * indicates p ≤0.05, ** p≤0.01, **** indicates p≤0.0001. # indicates p≤0.05, ## p≤0.01, ### p≤0.001.

In light of the observed attenuated hepatic inflammation in *Ldlr^-/-^* mice following chronic-binge ethanol feeding, our study sought to elucidate whether this reduction in inflammation is associated with a lower susceptibility to liver injury. Hence, we measured plasma alanine transaminase (ALT) levels, which were similar between *Ldlr^-/-^* and wild type mice (**Figure 5A**). Furthermore, we measured levels of thiobarbituric acid reactive substance (TBARS), a marker of oxidative stress, which is associated with hepatocellular damage, in the livers of mice subjected to chronic-binge ethanol feeding. No significant difference in TBARS levels was observed in livers of wild type and *Ldlr^-/-^* mice (**Figure 5B**). Next, although the chronic-binge ethanol feeding model to wild type mice is too mild and not sufficient to induce severe hepatic fibrosis, we assessed Sirius Red staining and hepatic expression of fibrosis-related genes to evaluate the effect of *Ldlr*-deficiency on alcohol-induced liver fibrosis. Interestingly, immunohistochemical scoring and detection of Sirius Red revealed that ethanol-fed *Ldlr^-/-^* mice developed increased collagen deposition compared to wild type mice (**Figures 5C–E**). In line, *Ldlr^-/-^* mice had significantly increased hepatic mRNA levels of *Tgfb*, *Col1a1*, and *Col3a1* than wild type mice after chronic-binge ethanol feeding (**Figures 5F–H**). These data suggest that systemic dyslipidemia in *Ldlr*-deficient mice enhances the initiation of ethanol-induced liver fibrosis development compared with wild type mice.

### *Ldlr^-/-^* mice do not show altered ethanol metabolism

**Figure 6:**
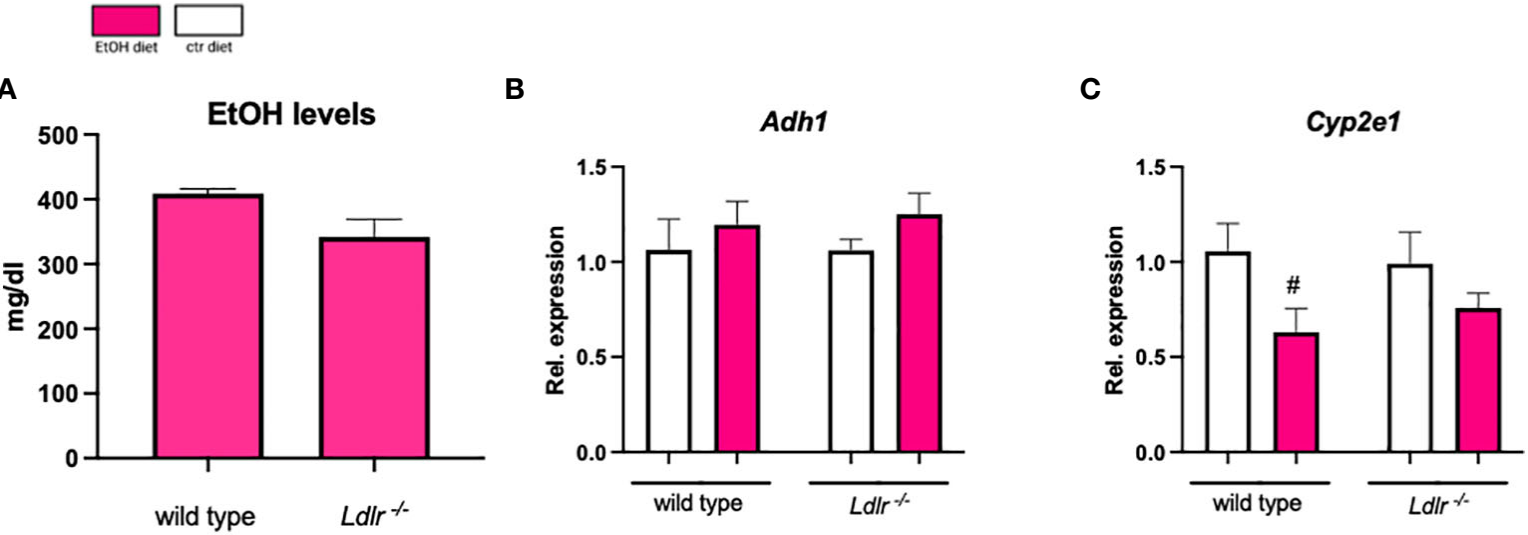
*Ldlr^-/-^* mice do not show alterations in ethanol absorption and metabolism. **(A)** Plasma ethanol levels at the study endpoint in ethanol-fed C57BL/6J and *Ldlr^-/-^* mice. **(B, C)** mRNA levels of indicated genes (*Adh1, Cyp2e1*) in livers of ethanol- and control-fed female C57BL/6J and *Ldlr^-/-^* mice, assessed by qPCR. Data are shown relative to the isocaloric control-fed mice and normalized to *18S*. Data shown as mean ± SEM of n=4-16/group. # indicates significant differences between isocaloric control-fed and ethanol–fed mice. # indicates p ≤0.05.

To assess whether our observed changes in the hepatic inflammatory and fibrotic response are related to differences in ethanol metabolism, plasma ethanol levels and expression of hepatic genes important for ethanol metabolism were measured. Plasma ethanol levels (**Figure 6A**), as well as hepatic mRNA levels of *Adh1* and *Cyp2e1*, did not reveal differences between wild type and *Ldlr^-/-^* mice after ethanol feeding (**Figures 6B, C**). These data suggest that *Ldlr*-deficiency does not affect ethanol metabolism in the chronic-binge ethanol feeding model.

## Discussion

Chronic alcohol consumption mostly results in the development of alcohol-associated liver disease, ranging from steatosis to steatohepatitis, cirrhosis, and hepatocellular carcinoma. Since no interventions other than abstinence from alcohol are currently available and the cause for interindividual differences in the severity and progression of alcohol-related liver disease remains poorly understood, there is an urgent need to identify potential risk factors contributing to disease progression. Considering dysregulated lipid metabolism plays a significant role in all stages of the disease, we investigated the influence of pre-existing dyslipidemia on the development of alcohol-related liver disease. Our analyses revealed that chronic-binge ethanol feeding elevated systemic triglyceride levels in *Ldlr*-deficient mice, thereby affecting hepatic fibrosis initiation.

While we did not observe any effects of ethanol intake on systemic cholesterol levels, a study by *Wang et al.* reported that alcohol diet resulted in increased amounts of cholesterol in circulation due to downregulation of hepatic *Ldlr* expression, thereby lowering cholesterol uptake by the liver (13). Since experimental rats receiving Lieber-De Carli diet for 4 weeks were used, the difference in the animal model might explain any discrepancies observed in our study. Importantly, we found that ethanol consumption further raises high plasma triglyceride levels in the absence of LDLR. Considering that hypertriglyceridemia is a risk factor for cardiovascular disease, our results highlight that ethanol consumption among affected individuals might not only lead to alcohol-related liver disease but could further increase the risk of developing conditions affecting the heart and blood vessels (24).

Intriguingly, our findings demonstrated a decrease in hepatic neutrophil influx in *Ldlr^-/-^* mice, accompanied by indications of increased collagen deposition. In line with previously described observations that increased systemic lipid levels are associated with the severity of fibrosis (25–27), our data corroborate that systemic dyslipidemia is a key factor contributing to the progression of alcohol-related liver disease into fibrosis. Mechanistically, it is known that excessive lipid levels result in elevated oxidative stress and the formation of reactive oxygen species, thereby promoting injury and cell death. Importantly, cell death, as well as oxidative stress itself, are associated with the transdifferentiation of quiescent hepatic stellate cells (HSCs) into an activated collagen-secreting state, thereby initiating fibrosis (28–30). As such, accelerated fibrosis development in ethanol-fed dyslipidemic *Ldlr^-/-^* mice might result from elevated lipid-induced cell death and oxidative stress. Importantly, in line with our experimental data, increased LDL levels, as occurs in *Ldlr^-/-^* mice, were recently described to be an independent predictor of the severity and degree of hepatic fibrosis in patients with alcohol-associated liver disease (31). In addition, Lpl plays a pivotal function in facilitating the transfer of lipids from the bloodstream to various tissues, and due to its involvement in the regulation of lipid metabolism and energy homeostasis, increased *Lpl* expression has been shown to aggravate NASH. A study conducted by *Teratani* and colleagues (32) established a positive correlation between *Lpl* expression in HSCs and the worsening of fibrosis during NASH, thereby potentially explaining our observations of increased *Lpl* expression and hepatic deposition of collagen in *Ldlr^-/-^* mice (32). Moreover, despite the fact that our TBARS assay did not reveal any difference between wild type and *Ldlr^-/-^* mice after ethanol feeding, we observed increased hepatic expression of *Cd36*, which enables the uptake of oxidized lipids that enhance HSC activation, thereby potentially contributing to enhanced fibrosis. Taken together, our data confirm that metabolic profiling of patients with excessive alcohol consumption will help to identify the risk of developing alcohol-related end-stage liver disease.

Besides elevated circulatory lipid levels and altered lipid metabolism, other factors might contribute to accelerated fibrosis initiation in ethanol-fed *Ldlr^-/-^* mice. Considering alcohol consumption causes dysbiosis and increased gut leakiness (33–35), lipid-induced changes in the microbiome or altered intestinal permeability might enhance hepatic fibrosis. Indeed, *Ldlr^-/-^* mice have been shown to compose a different microbiome than wild type mice (36, 37). Hence, the translocation of specific gut-derived bacterial components in *Ldlr^-/-^* mice might promote HSCs activation and lead to more severe fibrosis after ethanol feeding (38–40). In addition, the intestinal microbiome affects bile acid metabolism and composition, which are involved in liver disease progression (41, 42). More specifically, secondary unconjugated bile acids have been shown to induce the expansion of HSCs, thereby promoting the progression of liver fibrosis (43, 44). Therefore, one might speculate that a modified bile acid composition is responsible for the increased collagen deposition in *Ldlr^-/-^* mice after ethanol diet. Further studies are needed to unravel the contribution of an altered microbiome and/or bile acid pool to aggravated collagen deposition in ethanol-fed dyslipidemic *Ldlr^-/-^* mice. Importantly, to exclude that our findings indicating enhanced systemic triglyceride levels and accelerated fibrosis after ethanol feeding during pre-existing dyslipidemia are restricted to *Ldlr*-deficient mice, confirmational studies using another mouse model should be undertaken, such as using *ApoE^-/-^* mice. Both *Ldlr^-/-^* mice and *ApoE^-/-^* mice exhibit impaired lipid metabolism, but the key distinction lies in the specific aspects of lipid clearance, resulting in elevated plasma cholesterol levels and altered lipid profiles. More precisely, *Ldlr*-deficiency is associated with increased LDL levels, while *ApoE*-deficiency is characterized by elevated levels of circulatory VLDL (45). Given that *ApoE^-/-^* mice also exhibit severe hypercholesterolemia and have more pronounced impaired immunoregulatory functions than *Ldlr^-/-^* mice one can speculate that ethanol feeding to *ApoE^-/-^* mice might further enhance dyslipidemia and steatohepatitis. Besides dyslipidemia in these models using genetically modified mice, it would be interesting to investigate ethanol-induced consequences during impaired lipid metabolism and systemic dyslipidemia within the context of wild type mice, i.e. combining a high-fat diet with ethanol supplementation. As such, a recent study by Chang et al. (46) introduced an experimental model that induces severe steatohepatitis through the administration of a high-fat diet for 3 days or 3 months combined with ethanol binge. While ethanol administration was shown to increase free fatty acid levels in the serum and liver, data describing triglyceride and cholesterol levels were lacking. Yet, this model seems to represent a suitable framework to examine the impact of ethanol on plasma and hepatic lipid levels in the presence of pre-existing dysregulated lipid metabolism in the setting of wild type mice.

In summary, we show that chronic-binge ethanol diet during systemic dyslipidemia in *Ldlr^-/-^* mice accelerates elevated plasma triglycerides and contributes to an early activation of a fibrotic response. Given the increased consumption of ethanol-containing beverages (47), the doubling of the prevalence of dyslipidemia between 2009 and 2019, and that familial hypercholesterolemia, which is caused by inherited mutations in the LDLR gene, affects 34 million people worldwide, our present study has important clinical implications (48, 49). Our results indicate that alcohol consumption by people with dyslipidemia or familial hypercholesterolemia may lead to more severe alcohol-related liver disease, besides enhancing their risk for cardiovascular complications. In addition, since *Ldlr^-/-^* mice display a human-like lipid profile with significant LDL-C in circulation compared to C57Bl/6J mice, our current data showing the development of ethanol-induced fibrosis in *Ldlr^-/-^* mice in the chronic-binge ethanol feeding provide evidence for a novel murine model for studying alcohol-associated liver disease (18).

## Data availability statement

The raw data supporting the conclusions of this article will be made available by the authors, without undue reservation.

## Ethics statement

The animal study was reviewed and approved by Animal Ethics Committee of the Medical University of Vienna and the Austrian Federal Ministry of Education, Science and Research (License number: 2022-0.574.136).

## Author contributions

CH performed the studies, acquired and analyzed the data, and wrote and edited the manuscript. DR, BS provided technical assistance and acquired part of the data. TH designed and performed the studies, analyzed the data, and wrote and edited the manuscript. All authors contributed to the article and approved the submitted version.
